# Supplementary material for: High-Density Lipoprotein Cholesterol and Cognitive Function in Older Korean Adults Without Dementia: Apolipoprotein E4 as a Moderating Factor
Source: Nutrients. 2025 Jul 14;17(14):2321. doi: 10.3390/nu17142321 (PMC12297950; doi:10.3390/nu17142321)
Supplement: Supplementary file 1 [file nutrients-17-02321-s001.zip › nutrients-3655362-supplementary.pdf]

Supporting Online Content

Table S1 Results of the multiple linear regression analyses of the association between the HDL-cholesterol level and cognitive decline according to APOE4 subgroup with additional adjustment for MNA total score

Table S2 Results of the multiple linear regression analyses of the association between the stratified HDL-cholesterol level and cognitive decline according to APOE4 subgroup with additional adjustment for MNA total score

Table S1 Results of the multiple linear regression analyses of the association between the HDL-cholesterol level and cognitive decline according to APOE4 subgroup with additional adjustment for MNA total score

	B	95% CI	β	*P*
TS				
Overall				
Model [a]	0.130	0.006-0.255	0.107	0.041
APOE4-negative				
Model [b]	0.009	-0.132-0.151	0.007	0.897
APOE4-positive				
Model [b]	0.590	0.387-0.793	0.654	<0.001
EMS				
Overall				
Model [a]	0.116	0.038-0.194	0.158	0.004
APOE4-negative				
Model [b]	0.056	-0.033-0.145	0.076	0.215
APOE4-positive				
Model [b]	0.381	0.225-0.537	0.591	<0.001

Abbreviations: B, regression coefficient; CI, confidence interval; β, standardized beta; P, p-value; APOE4, apolipoprotein E ε4 allele; MNA, mini nutritional assessment; EMS, episodic memory score; TS, total score of the Consortium to Establish a Registry for Alzheimer's Disease; VRS, vascular risk score, PASE, physical activity scale for the elderly; LDL

[a] The model included age, sex, APOE4, education, clinical diagnosis, VRS, PASE total score, albumin, fasting glucose, LDL-cholesterol, and MNA total score as covariates.

[b] The model included age, sex, education, clinical diagnosis, VRS, PASE total score, albumin, fasting glucose, LDL-cholesterol, and MNA total score as covariates.

Table S2 Results of the multiple linear regression analyses of the association between the stratified HDL-cholesterol level and cognitive decline according to APOE4 subgroup with additional adjustment for MNA total score

	B	95% CI	β	*P*
TS				
Overall				
Model [a]				
High HDL-cholesterol	5.279	0.154-10.405	0.156	0.044
Medium HDL-cholesterol	0.391	-4.727-5.509	0.011	0.880
Low HDL-cholesterol	Reference			
APOE4-negative				
Model [b]				
High HDL-cholesterol	0.341	-3.938-4.620	0.010	0.875
Medium HDL-cholesterol	-1.069	-5.340-3.202	-0.030	0.622
Low HDL-cholesterol	Reference			
APOE4-positive				
Model [b]				
High HDL-cholesterol	17.041	9.017-25.064	0.654	<0.001
Medium HDL-cholesterol	11.592	3.824-19.360	0.454	0.005
Low HDL-cholesterol	Reference			
EMS				
Overall				
Model [a]				
High HDL-cholesterol	3.441	1.022-5.860	0.168	0.006
Medium HDL-cholesterol	1.884	-0.517-4.284	0.091	0.123
Low HDL-cholesterol	Reference			
APOE4-negative				
Model [b]				
High HDL-cholesterol	1.964	-0.718-4.646	0.097	0.150
Medium HDL-cholesterol	0.723	-1.954-3.400	0.035	0.595
Low HDL-cholesterol	Reference			
APOE4-positive				
Model [b]				
High HDL-cholesterol	11.529	5.910-17.148	0.619	<0.001
Medium HDL-cholesterol	9.542	4.102-14.982	0.523	0.001
Low HDL-cholesterol	Reference			

Abbreviations: B, regression coefficient, CI, confidence interval; *β*, standardized beta; P, p-value; APOE4, apolipoprotein E ε4 allele; EMS, episodic memory score; TS, total score of the Consortium to Establish a Registry for Alzheimer's Disease; VRS, vascular risk score; PASE, physical activity scale for the elderly; MNA, mini nutritional assessment.

[a] The model included age, sex, APOE4, education, clinical diagnosis, VRS, PASE total score, albumin, fasting glucose, LDL-cholesterol, and MNA total score as covariates.

[b] The model included age, sex, education, clinical diagnosis, VRS, PASE total score, albumin, fasting glucose, LDL-cholestero l, and MNA total score as covariates.
